# High levels of IgG3 anti ICB2-5 in *Plasmodium vivax*-infected individuals who did not develop symptoms

**DOI:** 10.1186/1475-2875-12-294

**Published:** 2013-08-27

**Authors:** Fernanda G Versiani, Maria EM Almeida, Gisely C Melo, Francivaldo OL Versiani, Patrícia P Orlandi, Luís André M Mariúba, Leidiane A Soares, Luciana P Souza, Antonio A da Silva Balieiro, Wuelton M Monteiro, Fabio TM Costa, Hernando A del Portillo, Marcus VG Lacerda, Paulo A Nogueira

**Affiliations:** 1 Instituto Leônidas e Maria Deane - Fiocruz, Rua Teresina 476, 69057-070 Manaus, AM, Brazil; 2 Universidade Federal do Amazonas, Av. General Rodrigo Octávio Jordão Ramos, 3000, Campus Universitário, Coroado I, Manaus, Amazonas, Brazil; 3 Fundação de Medicina Tropical Heitor Vieira Dourado, Gerência de Malária, Av. Pedro Teixeira, 25, Dom Pedro, 69040-000 Manaus, AM, Brazil; 4 Universidade do Estado do Amazonas, Av. Pedro Teixeira, 25, Dom Pedro, 69040-000 Manaus, AM, Brazil; 5 Departamento de Genética, Evolução e Bioagentes, Instituto de Biologia, Universidade de Campinas (UNICAMP), Campinas, SP, Brazil; 6 ICREA at Barcelona Centre for International Health Research (CRESIB) Rosselló, 132, 4th floor, 08036 Barcelona, Spain

**Keywords:** Malaria, *Plasmodium vivax*, Merozoite surface protein-1, Clinical protection, IgG3

## Abstract

**Background:**

*Plasmodium vivax* has the potential to infect 2.85 billion individuals worldwide. Nevertheless, the limited number of studies investigating the immune status of individuals living in malaria-endemic areas, as well as the lack of reports investigating serological markers associated with clinical protection, has hampered development of vaccines for *P. vivax*. It was previously demonstrated that naturally total IgG against the N-terminus of *P. vivax* merozoite surface protein 1 (Pv-MSP1) was associated with reduced risk of malarial infection.

**Methods:**

Immune response against Pv-MSP1 (N-terminus) of 313 residents of the Rio Pardo rural settlement (Amazonas State, Brazil) was evaluated in a cross-sectional and longitudinal follow up over two months (on site) wherein gold standard diagnosis by thick blood smear and rRNA gene-based nested real-time PCR were used to discriminate symptomless *Plasmodium vivax*-infected individuals who did not develop clinical symptoms during a 2-months from those uninfected ones or who have had acute malaria. The acquisition of antibodies against Pv-MSP1 was also evaluated as survival analysis by prospective study over a year collecting information of new malaria infections in surveillance database.

**Results:**

The majority of *P. vivax*-infected individuals (52-67%) showed immune recognition of the N-terminus of Pv-MSP1. Interesting data on infected individuals who have not developed symptoms, total IgG levels against the N-terminus Pv-MSP1 were age-dependent and the IgG3 levels were significantly higher than levels of subjects had acute malaria or those uninfected ones. The total IgG anti ICB2-5 was detected to be an important factor of protection against new malaria vivax attacks in survival analysis in a prospective survey (p = 0.029).

**Conclusions:**

The study findings illustrate the importance of IgG3 associated to 2-months of symptomless in *P. vivax* infected individuals and open perspectives for the rationale of malaria vaccine designs capable to sustain high levels of IgG3 against polymorphic malaria antigens.

## Background

*Plasmodium vivax* is the most widespread species of human malaria parasite, and risk of *P. vivax* infection is higher compared to *Plasmodium falciparum*[1,2]. In Brazil, 99.8% of malaria cases are concentrated in the Amazon region with *P. vivax* being the most prevalent species (ca 85%) [3]. Although *P. vivax* is currently associated with non-life-threatening malarial infection, recent studies have reported similar complications and pathogenic mechanisms frequently observed in malaria caused by *P. falciparum*[4-6]. Drug resistance to commonly used anti-malarial drugs has also been reported worldwide [4,7-16] challenging the current view of *P. vivax* as a less harmful parasite and raising the need for developing an effective vaccine.

In hopes of reducing malaria morbidity and mortality, *Plasmodium* merozoite antigens have been proposed as targets for vaccine design [2]. Amongst this family of molecules, merozoite surface protein-1 (MSP1) is a 195-kDa glycoprotein abundant on the surface of merozoites and essential for merozoite development due to its involvement in erythrocyte invasion [17]. MSP1 attaches to the parasite membrane by a GPI anchor and associates with other merozoite molecules (e g, MSP6 and MSP7), forming a multicomplex protein [18]. MSP1 displays a highly polymorphic N-terminus, yet a conserved C-terminus region [17,19,20]. The *Pvmsp1* gene consists of six highly polymorphic domains (called polymorphic blocks) flanked by fairly conserved sequences (two, four and five blocks) [19] as interspecies conserved blocks called ICBs [20] and one conserved domain (CB-3).

Many studies have indicated that MSP1 is highly immunogenic in natural malarial infections and often associated with parasite exposure [21-26]. As such, it has considerable potential as a candidate target for vaccine design and/or clinical trials [27-32]. Although short-lived, *P. vivax* MSP1 (referred to as Pv-MSP1) humoral immune response has been shown to be mostly against the polymorphic domains [27,28,33-35]. In individuals clinically protected from malaria, high levels of antibodies against a polymorphic domain in the N-terminus of Pv-MSP1 [30].

The current study identified occurrence of symptomless *Plasmodium vivax*-infected individuals during a two-months follow-up at the Rio Pardo rural settlement (Amazonas State, Brazil), and performing humoral immune response analysis (total IgG and subclasses) against Pv-MSP1.

## Methods

### Selected area and population

**Figure 1:**
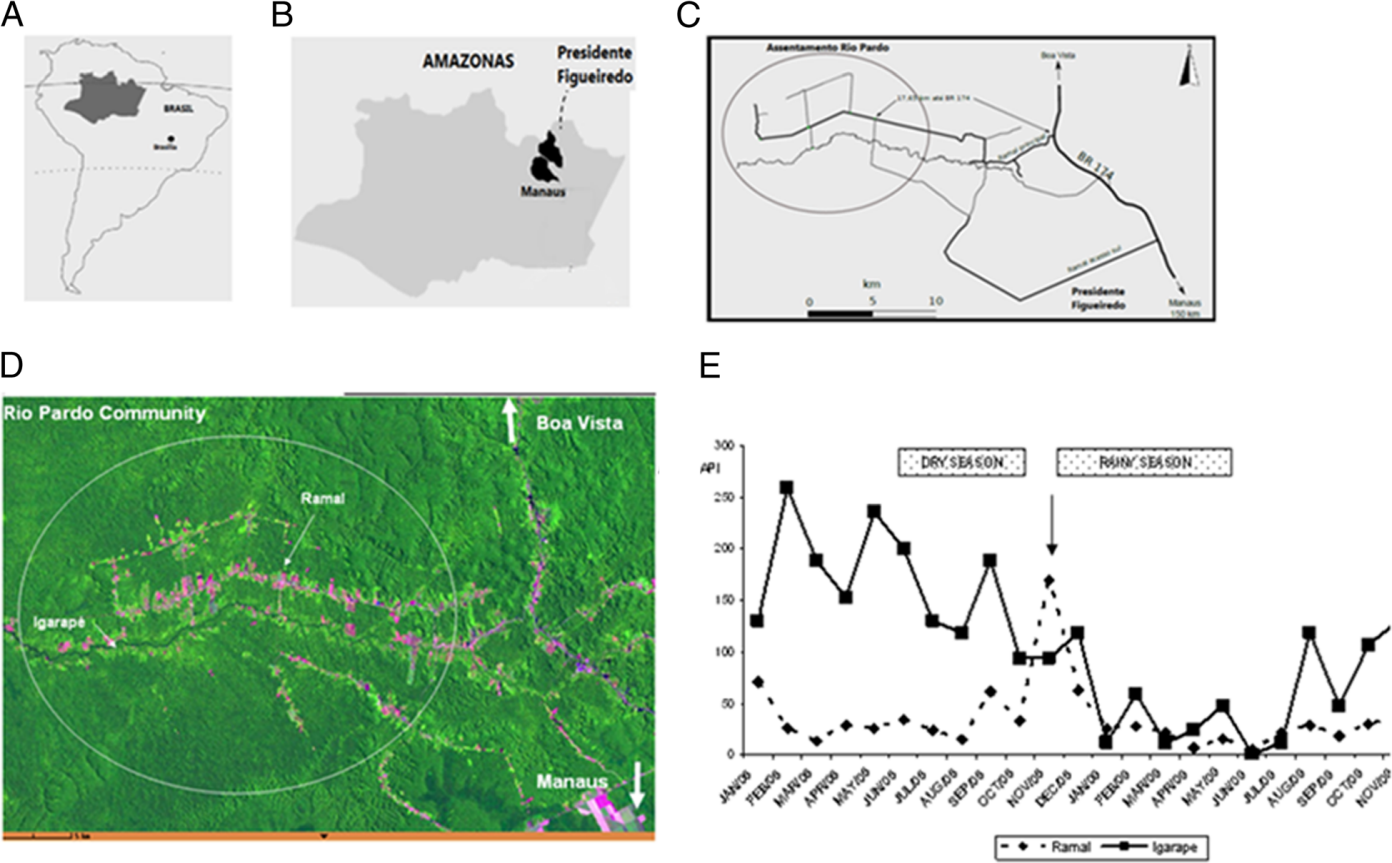
**Rio Pardo settlement location. (A-B)** The study took place in an agricultural settlement of Rio Pardo, in the northeast region of Amazonas State, Brazil. **(C)** Main access of community of Rio Pardo is via a paved road (BR-174) that connects the states of Amazonas and Roraima. **(D)** The settlement is comprised of households located along both sides of unpaved roads, which is a typical deforestation pattern in the Amazon [33]. Rio Pardo outline by Sylvain JM Desmoulière – ILMD. **(E)** The temporal distribution of API of *P. vivax* in the Ramal and Igarapé areas occurred in the period, with the rainy season well defined: the rainy season (November–May) and the dry season (June–October). The annual mean temperature was 31°C and average annual rainfall was 2,000 mm per year. Arrow: indicates cross-sectional study period.

At the Rio Pardo rural settlement (Figure 1) an agricultural settlement of Rio Pardo, Presidente Figueiredo municipality, in the northeast region of Amazonas State, Brazil. The rural community of Rio Pardo is located roughly 160 km from Manaus, the capital of the state. Main access is via a paved road (BR-174) that connects the states of Amazonas and Roraima. These unpaved roads have a main road that is connected perpendicularly to multiple side roads that are surrounded by tropical rain forest. The area of Rio Pardo consists of six side roads (eclipse). The settlement also includes a riverine community, where inhabitants live 1.5 km from the Rio Pardo stream margins. Inhabitants rely on subsistence farming and fishing along the Rio Pardo stream. The annual mean temperature was 31°C and average annual rainfall was 2,000 mm per year. Housing quality is poor, thereby rendering ineffective indoor residual spraying of insecticides against the mosquito vectors of malaria. Deforestation is common, although decreasing production has been faced in exploration areas. Health care access is extremely limited in the area, and only one health centre handles all diagnoses of suspected malaria cases by means of Giemsa-stained thick blood smears.

**Figure 2:**
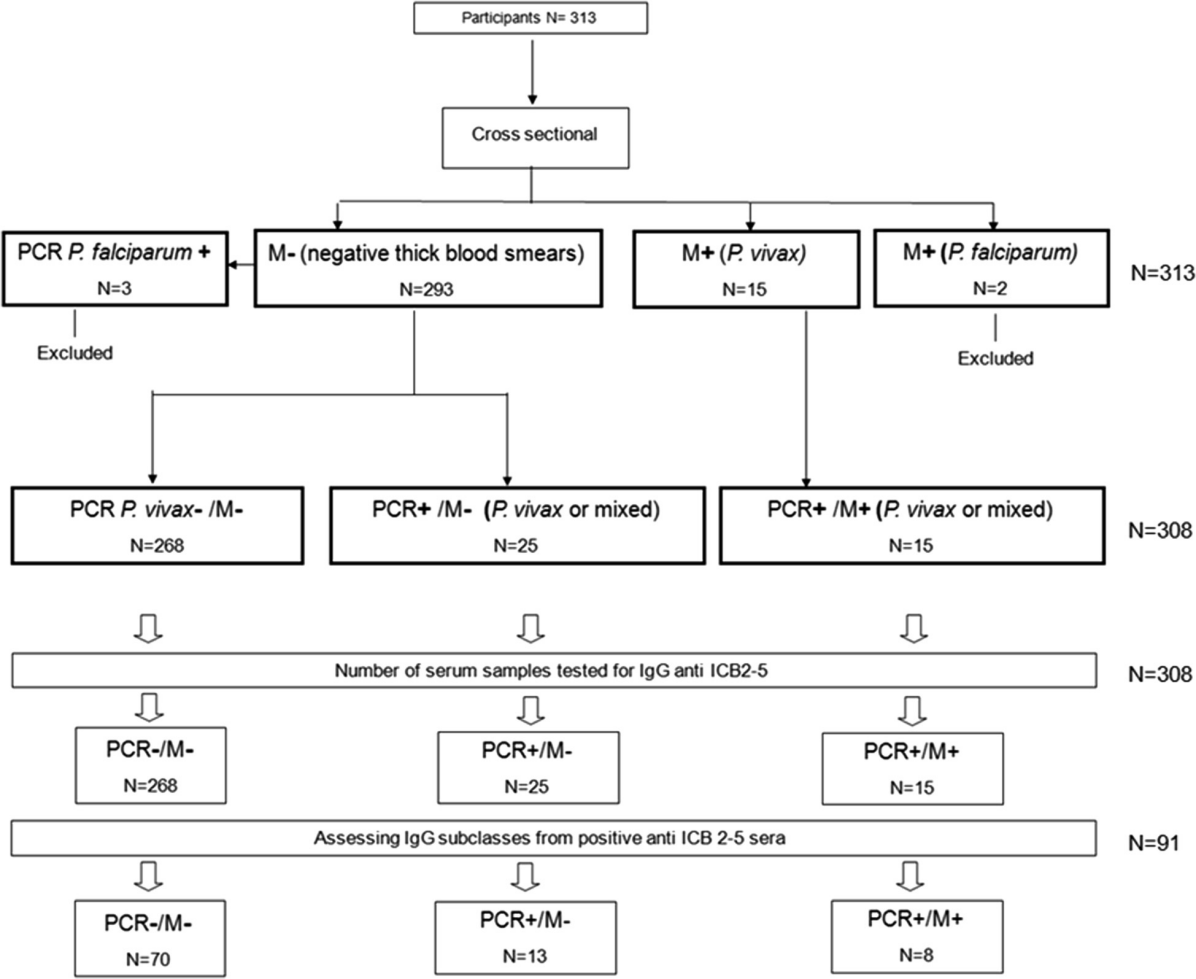
**Participant flow diagram.** The 313 individuals included in the study were classified into major groups (*M-* or *M+*) and further sub classified into three minor groups (*PCR-/M-, PCR+/M-* or *PCR+/M+*). For serology of total IgG and subclass, two patent *P. falciparum* mono-infection (N = 2) and *P. falciparum* mono-infection by PCR (N = 3) were excluded. Three hundred eight samples from the minor groups were used: *PCR+/M-* (N = 25), twenty five *P. vivax* infected individuals (three with mixed infections) had no malaria symptoms over two-month follow-up. Fifteen subjects from the *PCR+/M +* group had acute malaria during the study. Two hundred sixty eight were uninfected (*PCR-/M-*) at cross sectional. For subclass analysis, sera had sufficient amounts and that respond with total IgG anti ICB2-5 were evaluated, (N = 70) of *PCR-/M-*; (N = 13) of *PCR+/M-* and (N = 8) *PCR+/M +* .

In a census conducted from September to October 2008, five hundred nineteen inhabitants were identified, of which 51.4% lived along unpaved roads organized in a ‘fishbone’ pattern (a typical deforestation pattern for Amazon settlements) [36] and 48.6% lived in the Igarapé area located 1.5 km from the stream margins of the riverine community. Annual Parasitemic Index related to *P. vivax* was similar in both areas during the study period (Figure 1). Twenty individuals were excluded from the study due to double registration and one hundred eighty six samples were discarded due to poor DNA extraction or absence of serum samples. After applying these exclusion criteria, 313 individuals were included in the study (Figure 2).

### Study design

In November 2008, a cross-sectional study was performed in order to collect blood samples to determine malarial infection by Giemsa-stained blood thick smears and real-time PCR and for serological studies to evaluate humoral responses of individuals of the riverine community. At this time, a standardized questionnaire containing demographic, clinical and epidemiological data (number of previous malaria episodes, presence of signs and symptoms) was applied by trained and calibrated interviewers. After that, a longitudinal follow up (on site) was performed over two months and the participants continued to be interviewed and examined. Based on the results of Giemsa-stained thick blood smears obtained at cross sectional, the participants were initially classified into major groups: (*M-*) negative or (*M+*) positive thick blood smear, for *P. vivax* or *P. falciparum* infection. Next, rRNA gene-based nested PCR assay was performed to identify *P. vivax, P. falciparum* or mixed (*P. vivax* and *P. falciparum*) infections and the major were classified in minor subgroups: (*PCR+/M-*) positive mixed or *P. vivax* PCR and negative Giemsa-stained thick blood smears that showed no clinical symptoms of malaria during the two months of follow-up; (*PCR+/M+*) individuals positive for mixed or *P. vivax* PCR that were positive *P. vivax* for Giemsa-stained thick blood smears at cross sectional survey and had acute malaria. All subjects received anti-malarial drugs according to the practical guide for malaria treatment in Brazil [37]. *(PCR-/M-)* negative individuals, who had no infection at cross sectional survey and individuals who were PCR positive or had malaria exclusively by *P. falciparum* were excluded from the study. Over 360 days, a prospective study was carried out and information about new malaria infection by *P. vivax* was collected through SIVEP-malaria database.

### Sample collection and malarial infection diagnosis

Thick blood smears for malaria diagnosis were collected and read by a local microscopist as recommended by the Brazilian Ministry of Health [37]. The slides were sent to a central laboratory and reviewed by an experienced microscopist, who confirmed the diagnosis.

Five ml peripheral blood was harvested by vein puncture, processed and stored at -20°C. DNA was extracted using a QIAGEN kit according to the manufacturer’s protocol. Electrophoresis was performed on a 0.6% agarose gel in 0.5X TBE buffer to confirm successful DNA extraction. Real-time PCR diagnoses were performed by means of a multiplex reaction as described elsewhere [38,39]. All primers and probes were purchased from Applied Biosystems™. To discriminate between *Plasmodium* species, probes were constructed using a VIC or FAM dye flag for *P. vivax* or *P. falciparum*, respectively. TAMRA quencher was used for both probes. For standardization of the real-time PCR protocol, 30 malaria negative and 50 malaria positive samples from the FIOCRUZ-ILMD bank were used to optimize the test.

### Recombinant protein production

The recombinant protein ICB2-5 (representing the N-terminus of Pv-MSP1) comprises three conserved blocks (Blocks 1, 3, and 5) and two variable blocks (Blocks 2 and 4) from the MSP1 protein of *Belem* strain as glutathione S-transferase (GST) fusion proteins [27-30,33]. As a control, GST alone was also produced. GST and *P. vivax* recombinant protein were purified using glutathione-sepharose 4B columns (Amersham Pharmacia), and protein concentration was determined by Bio-Rad Protein Assay Kit I (Bio-Rad Laboratories, Inc) [27-30].

### Humoral immune response analysis

Naturally acquired IgG antibodies against Pv-MSP1 have been characterized elsewhere by ELISA [27]. Based on individual values for OD against recombinant proteins, all tests were done in duplicate. Firstly the average OD of ICB2-5 was calculated to exclude reactivity against GST. For each individual, the GST cut offs were calculated as average OD of GST adding two Standard Deviation (SD) values. Thus, a serum was considered positive if ICB2-5 OD minus individual GST cut-offs was greater than zero. Based on population variation against ICB2-5, the ICB2-5 cut off were calculated as average OD of sera samples adding two SD values from 20 healthy individuals who had never suffered malaria.

Similarly, the positivity of serum to ICB2-5 was determined if the average OD of ICB2-5 was greater than ICB2-5 cut off. For each IgG subclass the ICB2-5 cut off was determined from same 20 healthy individuals who had never suffered malaria.

IgG subclasses were determined by ELISA using specific monoclonal antibodies for each isotype (Sigma, St. Louis, MO, USA) as described [27,30]. All sera were tested at dilutions of 1:100 in duplicate and monoclonal antibody binding was detected with peroxidase-conjugated anti-mouse immunoglobulin (Sigma).

### Statistical methods

Statistical analysis was performed using The R Project for Statistical Computing version 3.0.1. Normality was tested by Kolmogorov-Smirnov test. Logistic regression predicting positive vs. negative IgG anti ICB2-5 using non-infected subjects (PCR-/M- group) as reference was used to compare age, time of residence and time of last malaria attack. Differences in total IgG and subclasses levels were assessed between three groups (PCR-/M-; PCR+/M + and PCR+/M-) by one-way nonparametric Kruskal-Wallis test (first panel). Analysis to assess which group total IgG and subclasses levels were different was performed by non-parametric Dunn’s test for multiple comparisons (second panel). The significant differences (p < 0.05) were identified as letters “a” or “b”, intermediate level of antibodies was “ab”. The Kaplan-Meier survival analysis was performed to evaluate the probability of *P. vivax* infection over one year follow-up period (November 2008 to November 2009) between the serology groups negative and positive for ICB2-5. Individuals who were found infected in the cross-section period using Giemsa-stained thick blood smears were excluded from this analysis. P-values <0.05 were considered statistically significant.

### Ethical procedures

The study was approved by the Research Ethical Committee of the Federal University of Amazonas (Ethical Approval No. 3640.0.000.115-07). Informed written consent was obtained from all participants. For those under 18 years old, parents were instructed about the objectives of the study and signed together with the participant an informed consent. All patients tested positive in the thick blood smear during the cross-sectional study were treated according to the anti-malarial treatment guidelines from the Brazilian Ministry of Health.

## Results

### Recruitment and participant flow

From a census of more than 500 residents followed cross-sectional from September to November 2008, three hundred thirteen residents met inclusion criteria to participate of the current study. The epidemiological data from this human population were recorded through cross-sectional surveys and summarized in Table 1. A participant flow diagram for the study is shown in Figure 2.

**Table 1:** Summary of epidemiological results

**Characteristic**	**Result**
Gender	
Male (n(%))	180 (59)
Female (n(%))	124 (40.8%)
Total (n)	304*
Age (median (SD)) (Minimum-Maximum)	32.78 (SD 19.2592)
TR (years)	
0-5	99 (35.1%)
6-15	98 (34.8%)
> 15	85 (30.1%)
Total	282*
NI (n (%))	
0	22 (7.9%)
1-4	105 (37.5)
> 4	153 (54.6%)
Total	180*
LA (months)	
0-1	28 (9.0%)
2-3	30 (9.6%)
4-5	13 (4.2%)
6-12	23 (7.4%)
>12	181 (58.0%)
Never had malaria or didn’t know	37 (11.9%)

*information obtained trough interview, some individuals didn’t know the information.

TR-Time of residence in Rio Pardo settlement, *NI* Number of previous infections, *LA* Time in months since the last malaria attack, *INF* malaria infection. We consider statistically significant *p* values lower than 0.05. The differences in proportions were evaluated by chi-square (_X_^2^) test. For analysis between two continuous variable Spearman’s (SC).

Giemsa-stained thick blood smears and rRNA gene-based nested PCR assay to identify *P. vivax*, *P. falciparum* or mixed (*P. vivax* and *P. falciparum*) infections performed through cross-sectional surveys were summarized in Table 2. When the subjects were stratified based on diagnosis by thick blood smear (Figure 2), two hundred ninety six individuals were negative (*M-*) and fifteen individuals had *P. vivax* malaria during cross sectional survey (*M + P. vivax*). Two individuals had acute *P. falciparum* mono-infection and were excluded (Figure 2).

**Table 2:** Result for the methods used for diagnosis

**PCR**	**Microscopy analysis**				
	**Negative**	***Plasmodium vivax***	***Plasmodium falciparum***	**Mixed infection**	**Total**
Negative	268 (85.6%)	0 (0.0%)	0 (0.0%)	0 (0.0%)	268 (85.6%)
*P. vivax*	22 (7.0%)	14 (4.4)	0 (0.0%)	0 (0.0%)	36 (11.5%)
*P. falciparum*	3 (1.0%)	0 (0.0%)	2 (0.6%)	0 (0.0%)	5 (1.6%)
Mixed infection	3 (1.0%)	1 (0.3%)	0 (0.0%)	0 (0.0%)	4 (1.2%)
Total	296 (94.5%)	15 (4.8%)	2 (0.6%)	0 (0.0%)	313 (100%)

After diagnosis by rRNA gene-based nested PCR assay for *P. vivax* and *P. falciparum*, three individuals with *P. falciparum* mono-infection (*PCR P. falciparum +*) were excluded. From three hundred and eight individuals diagnosed by PCR, two hundred sixty eight individuals were uninfected at cross sectional because they were negative by microscopy examination and confirmed by PCR (*PCR-/M-*).

Further subclassification indicated that acute *P. vivax* infection was confirmed by microscopy examination and PCR in the fifteen individuals (*PCR+/M+)* of which one had acute *P. vivax* and *P. falciparum* infections*.* Twenty-five individuals whose diagnosis of *P. vivax* infections (which three with *P. vivax* and *P. falciparum* infections) was seen only in nested-PCR (*PCR+/M-*) showed no clinical symptoms of malaria over two-month follow-up (Figure 2).

### Assessing humoral response against the N-terminus of Pv-MSP1

**Figure 3:**
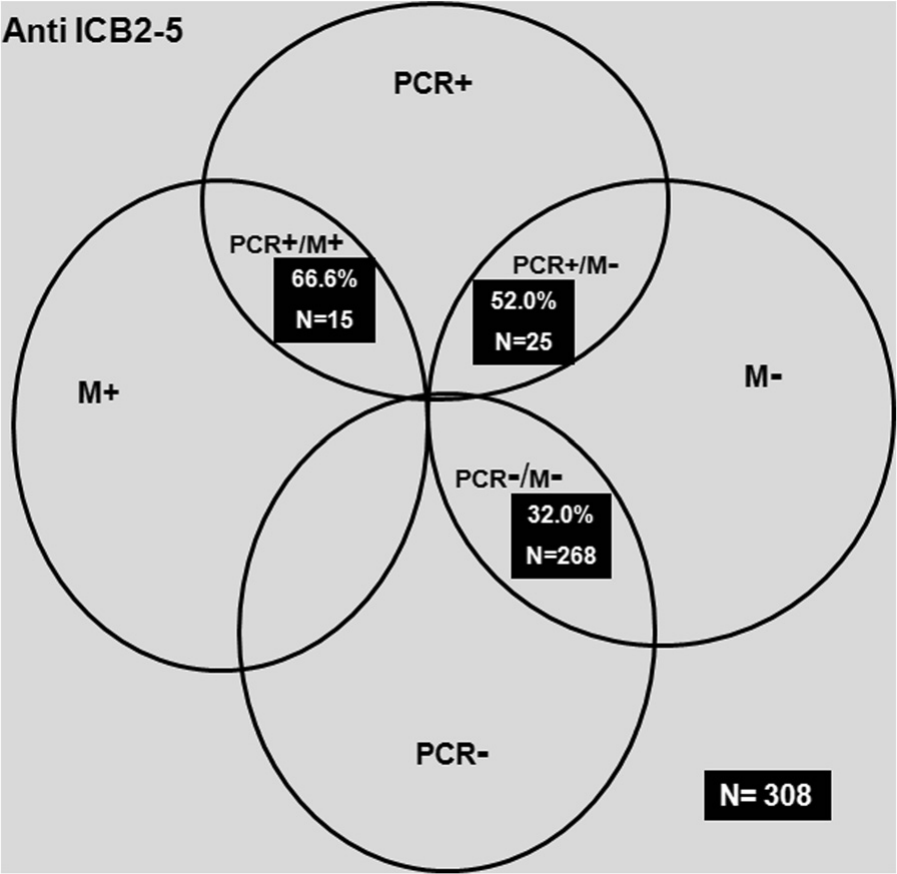
**Classification of the cross-sectional study.** The percentage of IgG responders to ICB2-5 were determined in 308 individuals classified into three minor groups (*PCR+/M+, PCR+/M-* or *PCR-/M-*).

Acquisition of natural antibodies against the N-terminus of Pv-MSP1 showed from all samples tested, one hundred nine individuals responded with IgG anti ICB2-5 (109/308), summarized in Figure 3. Analysis revealed that 66.6% of individuals from *PCR+/M+* (10/15) responded to ICB2-5, whereas among the subjects who did not have malaria infection until cross sectional survey (*PCR-/M-*), the frequency response was 32.0% (86/268). Importantly, frequency of responders to ICB2-5 from *PCR+/M-* was 52.0% (13/25) and slightly lower than *PCR+/M+* (not statistically significant).

**Figure 4:**
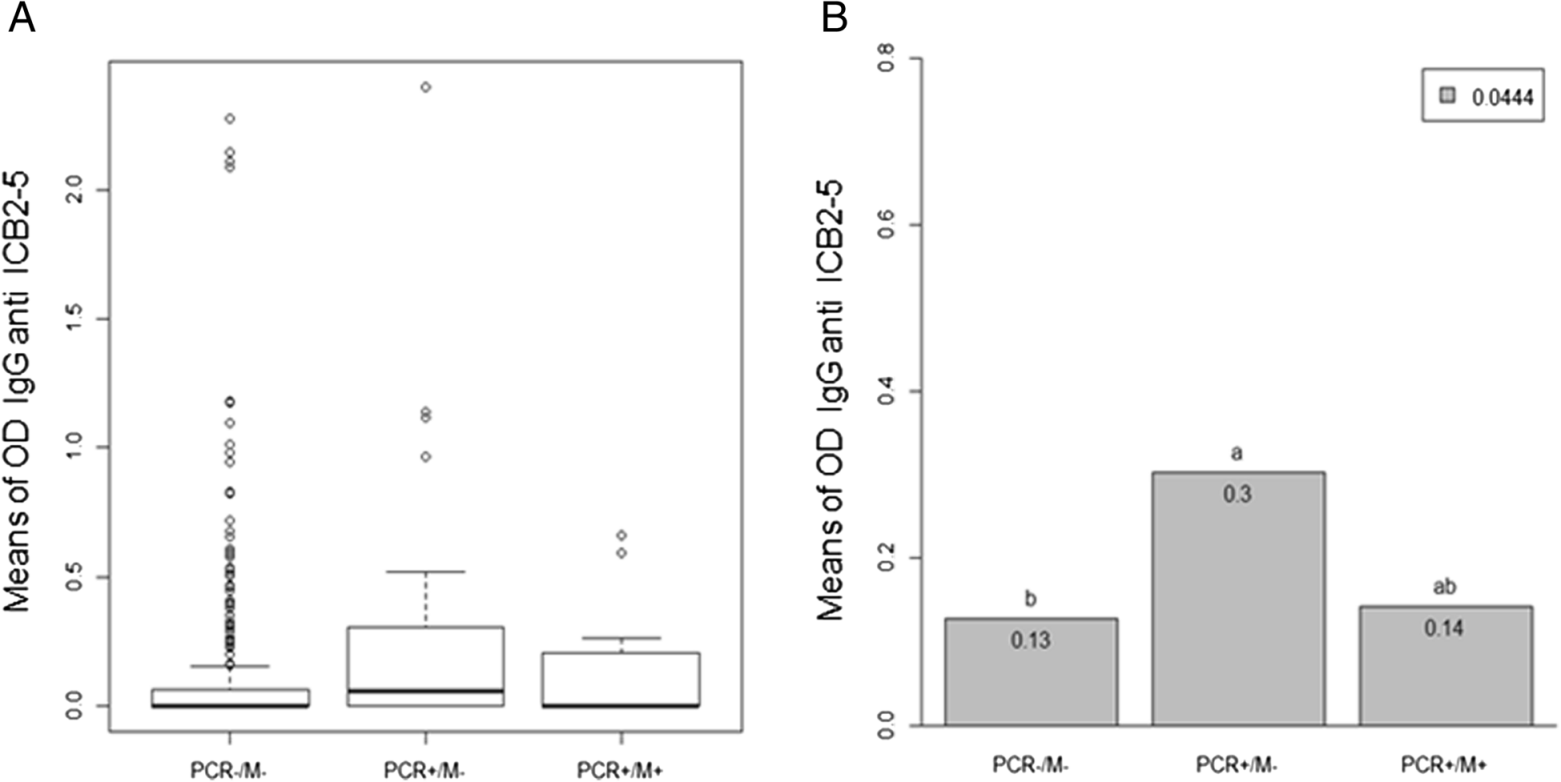
**Acquisition of IgG antibodies against the N-terminus of Pv-MSP1. (A)** Comparison of median of IgG anti ICB25 of the three minor groups is shown by one-way non-parametric Kruskal-Wallis test. **(B)** The mean level of IgG anti-ICB2-5 using non-parametric Dunn’s test for multiple comparisons. Statistically differences in the mean antibody at p < 0.05 is shown as letters “a” or “b”. Intermediate level of antibodies is shown as “ab”.

Comparing the median of IgG anti ICB25 by one-way non-parametric Kruskal-Wallis test, the levels of these antibodies were different among the three groups tested (Figure 4A). Using non parametric Dunn's test for multiple comparisonsto show differences as letters “a” or “b” at p < 0.05, the *PCR+/M-* group had more IgG anti ICB2-5 than uninfected individuals, whereas subjects with acute malaria had an intermediate level of antibodies “ab” (Figure 4B).

Using logistic regression to assess antibody prediction into groups and epidemiological data (Table 3), individuals of *PCR+/M-* had 2.45 more likely to have IgG anti ICB2-5 than reference group, *PCR-/M-* (P = 0.036). Analysing some epidemiological data, the presence of IgG antibodies ICB2-5 was solely dependent on the age (P = 0.003).

**Table 3:** Logistic regression predicting positive vs. negative IgG anti ICB2-5

**Variable**	**IgG**		**Odds ratio**	**CI 95%**	
	**Positive**	**Negative**			***P***
*Groups*					
PCR-/M-(Reference)	86 (81,13)^#^	181 (90,05)			
PCR+/M-	13 (12,26)	12 (5,97)	2.450	(1.06,5.69)	0.0360*
PCR+/M+	8 (6,60)	7 (3,98)	2.050	(0.68,6.11)	0.2000
*Age*	37.65 ± 1.92	30.48 ± 1.35	1.020	(1.01,1.03)	0.0020**
Time of residence	9.0 ± 3.4	7.5 ± 4.1	1.003	(0.999,1.0074)	0.4580
Time of last malaria attack	2 ± 5.1	2 ± 3.1	1.001	(0.997,1005)	0.983

Note: Variables of time (age time of residence and time of last malaria attack) are shown in years according to Mean ± Standard Error.

# in perenthesis: percentage per column.

‘*’ P-value < 0.05.

‘**’ P-value < 0.01.

**Figure 5:**
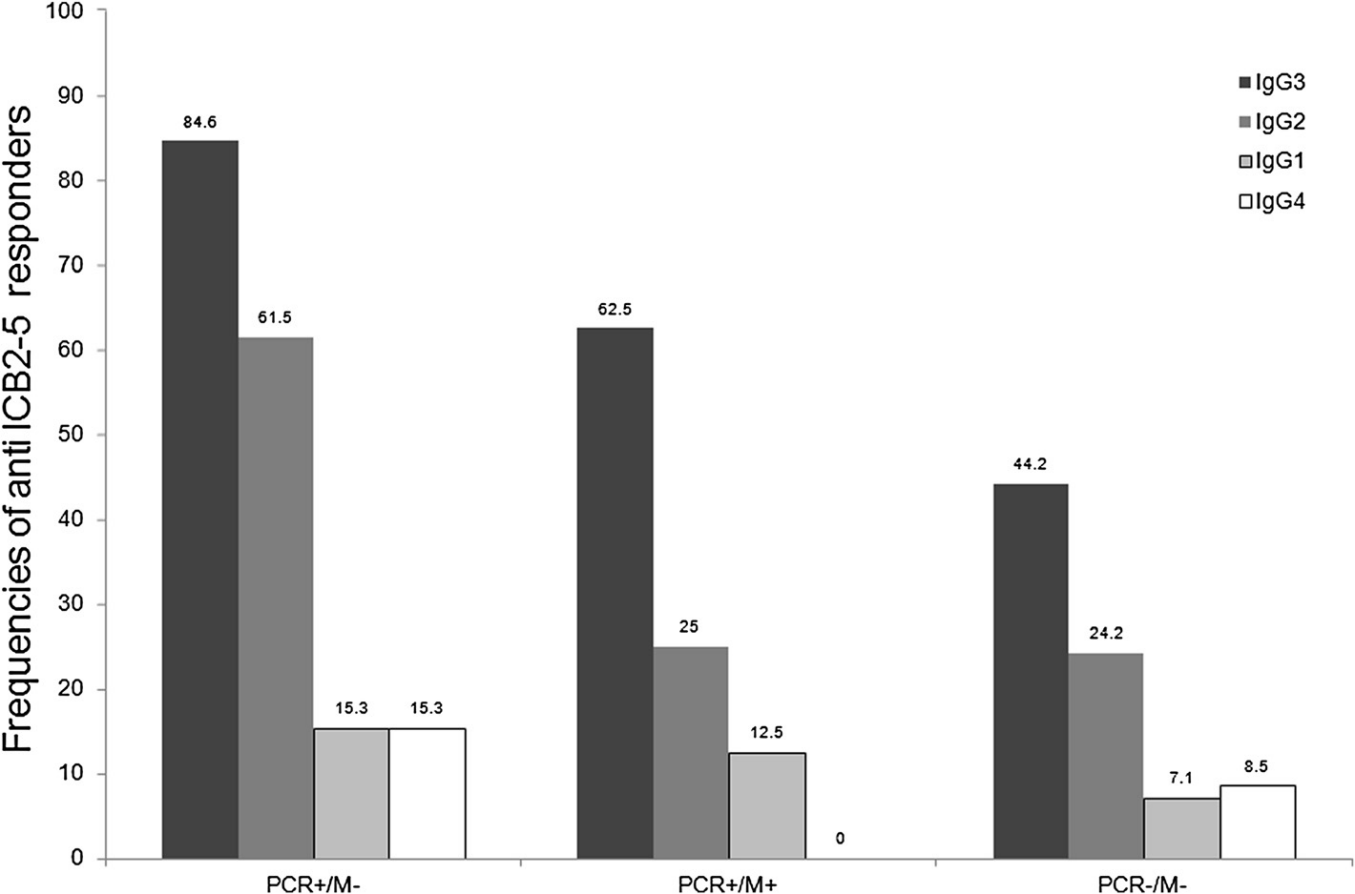
**Frequencies of responders with IgG subclasses to ICB2-5 between groups.** The IgG subclasses profiles to ICB2-5 were evaluated in ninety one individuals classified in three minor subgroups: *PCR+/M-* (N=13); *PCR+/M+* (N=8) and *PCR-/M-* (N=70).

The subclass analysis was performed only those that had sufficient amounts of serum (N = 91) and numbers of tested sera for subclasses each group is shown in Figure 2. The characterization of IgG subclass anti-ICB2-5 as a whole revealed that IgG3 anti-ICB2-5 was the most prevalent at 51.6% (47/91), IgG2 29.6% (27/91). Both IgG1 and IgG4 subclasses comprised 8.7% (8/91) each. When analyses were determined between groups, the IgG3 frequencies were increased in *PCR+/M-* 84.6% (11/13) and *PCR+/M +* 62.5% (5/8) and reduced 44.2% (31/70) in the uninfected group (Figure 5). The IgG2 frequencies increased only in infected groups, *PCR+/M-* 61.5% (8/13) remaining the same in both groups 25.0% (2/8) in *PCR+/M+* and 24.2% (17/70) in uninfected individuals (*PCR-/M-*). The frequencies of others subclasses were low.

**Figure 6:**
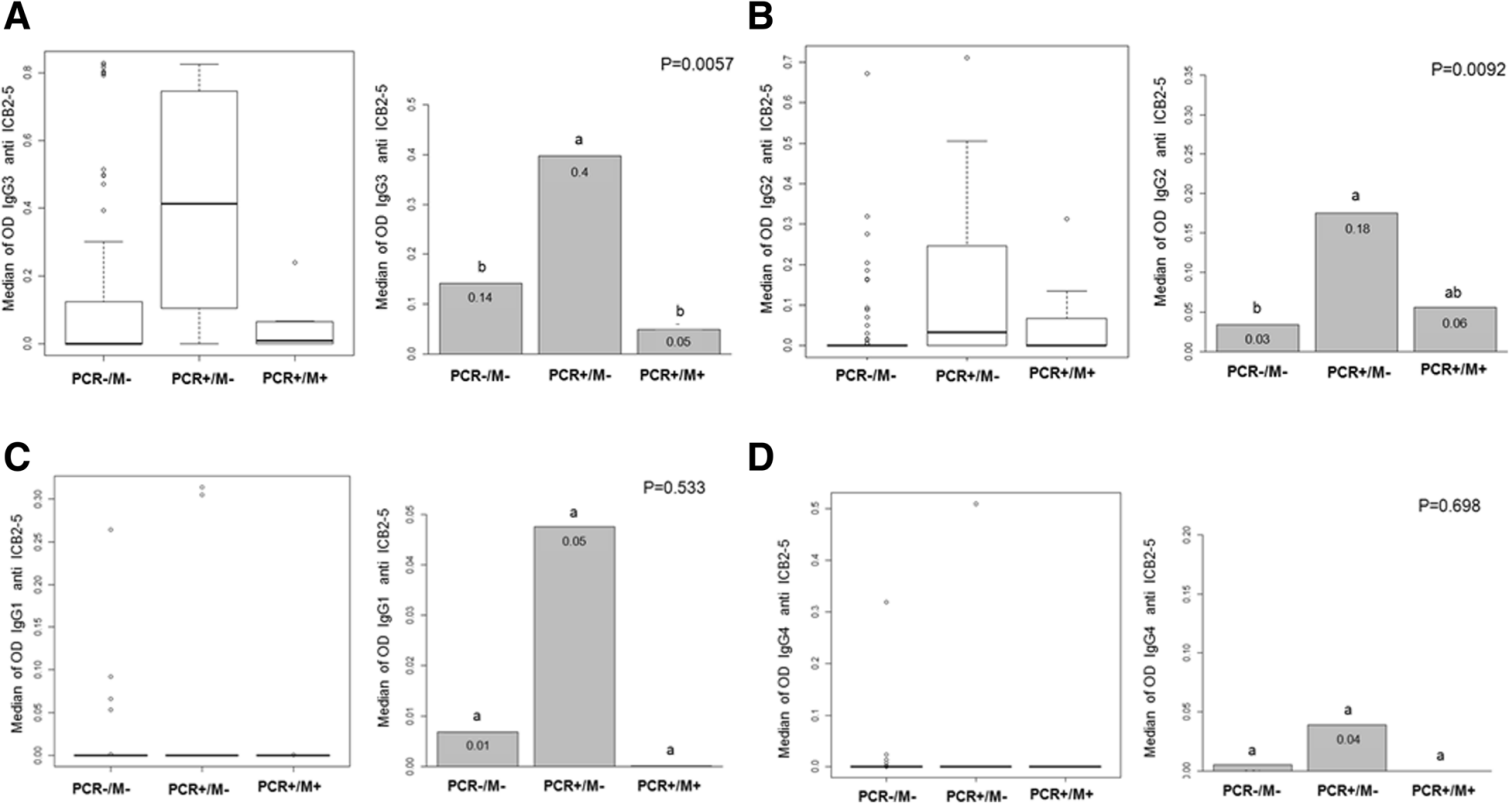
**Analysis of IgG subclass in responders to ICB2-5.** Differences in subclasses levels were assessed between groups. **A)** IgG3 levels against ICB2-5 of individuals from *PCR+/M-*; *PCR+/M +* and *PCR-/M-* were compared by one-way non-parametric Kruskal-Wallis test (first panel) to assess differences between groups. Analysis to assess which group the levels of IgG3 were different was performed by non-parametric Dunn's test for multiple comparisons (second panel). The significant differences (p < 0.05) were identified as letters “a” or “b”, intermediate level of antibodies was “ab”. The same analyses were performed for other subclasses levels against ICB2-5: **B)** IgG2; **C)** IgG1 and **D)** IgG4.

When the subclasses levels were assessed between groups by one-way non-parametric Kruskal-Wallis test, increased levels of IgG3 anti ICB2-5 in infected individuals who did not develop symptoms (*PCR+/M-*) were highlighted (Figure 6A). The non-parametric Dunn's test for multiple comparisons confirmed that IgG3 anti ICB2-5 levels in these individuals were significantly higher (p = 0.0057) than those with acute malaria (*PCR+/M+*) or uninfected (*PCR-/M-*).

Considering others IgG subclass, although there were some differences in IgG2 response between *PCR+/M- vs*. *PCR-/M-* (p = 0.0092) levels observed between groups were very low (Figure 6B). And still, the mean levels of IgG1 and IgG4 were not different in comparison between groups (Figure 6C-D).

**Figure 7:**
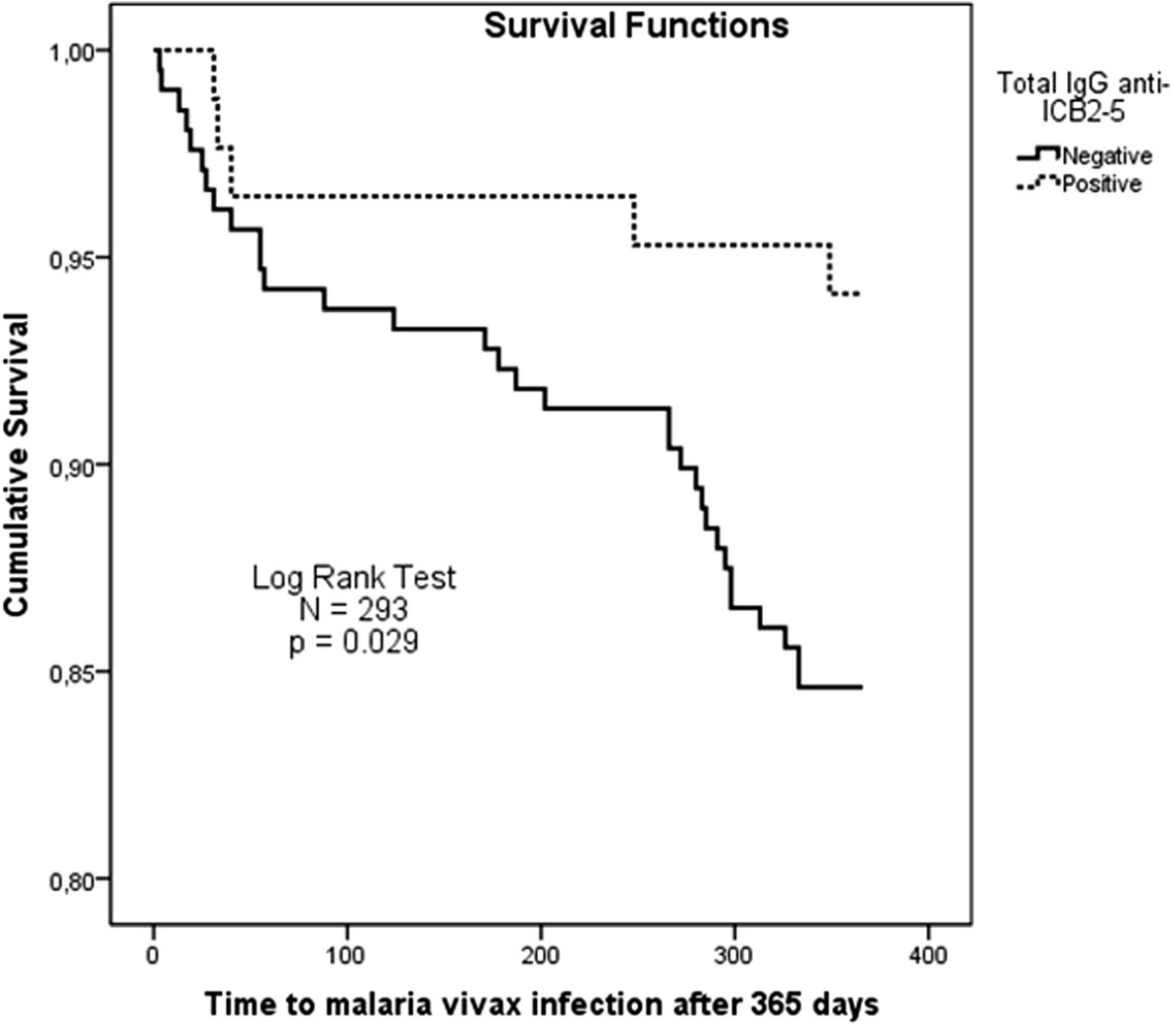
**Kaplan-Meier survival analyses.** The survival analysis was performed with two hundred ninety three (N = 293), because fifteen subjects who had acute malaria at cross sectional survey were excluded. For prospective study of 365-days individuals who responded with total IgG anti ICB2-5 at cross sectional survey had lower malaria attacks (P = 0,019; Log Rank Test).

The prevalence of antibodies for ICB2-5 was analysed as a determinant factor for protection against malaria attacks in a one-year follow-up. To this, a Kaplan-Meier survival analysis was performed with two hundred ninety three individuals from 313 participants (Figure 7). The total IgG anti ICB2-5 was detected to be an important factor of protection against malaria vivax attacks (p = 0.029). Unlike the total IgG, the low number of positive IgG3 anti ICB2-5 hampered survival analysis.

## Discussion

In the current study, antibody immune response against Pv-MSP1 was evaluated from a cross-sectional survey with 308 individuals living in hyperendemic areas. Higher levels of IgG3 against ICB2-5, corresponding to the N-terminus of MSP1 in *P. vivax*, were observed only in symptomless *Plasmodium vivax*-infected individuals.

Despite that recombinant ICB2-5 contains long stretches of amino acids that are conserved among the *P. vivax* haplotypes (*Belem* and *Salvador*), which explains the higher frequency of responders to this polypeptide in endemic areas in Brazil [20,27,29], it has been well established that these antibodies primarily recognize the variable domains (Blocks 2 and 4) of ICB2-5 [27,35]. However, Pv-MSP1 Block 2 is poorly immunogenic and achievement of clinical protection may be dependent on successive exposure of polymorphic blocks or eliciting short-lived antibody responses that also require frequent boosting as occurs in asymptomatic infections [35].

Investigation of total IgG response to the N-terminus of Pv-MSP1 revealed no significant differences between individuals of *PCR+/M-* and *PCR+/M+*. As both groups represent respectively, contingents of infected persons with parasitaemia undetectable in thick blood smears and without clinical signs of malaria and those with patent parasitaemia and clinical symptoms, these data suggest that total IgG against the N-terminus of Pv-MSP1 may not be the only predictor of clinical protection. Nonetheless, total levels of IgG against the N-terminus of Pv-MSP1 were higher in individuals without clinical symptoms of malaria, corroborating with previous study [30]. Moreover, whereas here IgG total levels were considered as a mean of *PCR+/M-* group, in previous study IgG total levels were analysed separately to each subject [30].

Analysis of antibody subclasses showed a stark difference in IgG3 levels of individuals from *PCR+/M-* in comparison to those had acute malaria, suggesting that higher levels of this subclass against the N-terminus of Pv-MSP1 could be related to absence of symptoms of malaria over two months of follow up. As parasitemia levels are detected only by real-time PCR and absence of symptoms of malaria does not allow determining since when these individuals were infected, it can be inferred that IgG3 dependent on continued exposure to the parasite could also be associated with this immune status.

Association between IgG3 antibodies and clinical protection from malaria was previously reported for *P. falciparum* antigens, such as MSP1_42_, MSP1_19_, AMA-1, MSP2 and MSP3 [22,23,40-44]. Similarly, IgG3 immune response to *P. falciparum* MSP1 (N-terminus) was associated with prolonged periods without malarial infection [45]. Moreover, the presence of IgG3 against Block 2 of Pf-MSP1 of the individuals would be held by asymptomatic infection confers a protective effect for extended periods [46]. IgG3 requires continuous stimulation to maintain effective levels of protection since IgG3 acts directly and indirectly to counter merozoite infection of red blood cells through the activation of monocytes [47-49].

Several studies have shown polymorphic antigens, including variants of the PfMSP1 Block-2, variants of the MSP-2, PvMSP5 frequently recognized by short-lived, species-specific antibodies [50-54]. It is possible that tandem, repeated, epitope sequences influence the ability to stimulate effective memory B-cell responses, unlike responses to antigens without repeat sequences (AMA1, EBA175, MSP2 or MSP119), in which responses are generally by both IgG1 and IgG3 [40,41,43,44,46,55-58]. According to conservation theory, the IgG subclass may be associated with the degree of conservation of the antigen, with conserved antigens preferentially inducing IgG1, and polymorphic antigens preferentially inducing IgG3, particularly if they contain repetitive polymorphic sequences [50].

The presence of high levels of IgG antibodies against the N-terminus of Pv-MSP1 was previously shown in asymptomatic individuals infected with *P. vivax*[30]. In this present study, information of new malaria infections was collected in Brazilian surveillance database over a year confirmed the Kaplan-Meier survival analysis for IgG total. However, the low number of positive IgG3 anti ICB2-5 hampered the Kaplan-Meier survival analysis for IgG3. Some factors could explain the lack of association of IgG3 in the survival analysis. It has been suggested that detectable levels of IgG3 may be particularly short-lived due to more rapid clearance of this subclass from the circulation [45,46,50,54]. There was a decay API related to *P. vivax* incidence during the surveillance period (Figure 1E). And still, the decrease in the number of positive IgG3 responders would be inherent to the immunoassay, even using monoclonal antibodies commonly used in Brazilian populations.

The immune status established by *P. vivax* subpatent infection and symptomless indicates that ICB2-5 (corresponding to haplotype *Belem*) may be a potential candidate antigen to consider for malaria vaccine development. Based on study findings, optimal immunity may require sustained high levels of IgG3 against polymorphic malaria antigens, which may require special strategies that need to be factored into the vaccine development process.

## Competing interests

The authors declare that they have no competing interests.

## Authors’ contributions

FGV carried out immunoassays. MESMA carried out expression of proteins. GCM, FOLV, LAS and LPS participated in the cross sectional study. PPO and LAMM carried out PCR. AASB performed the statistical analysis. WMM performed the prospective study accompanying the database. FTMC participated in the design of the study and manuscript. HAdP contributed reviewing data. MVGL contributed reviewing data and coordination. PAN participated in its design and coordination. All authors read and approved the final manuscript.
